# *In vitro* Neo-Genesis of Tendon/Ligament-Like Tissue by Combination of Mohawk and a Three-Dimensional Cyclic Mechanical Stretch Culture System

**DOI:** 10.3389/fcell.2020.00307

**Published:** 2020-06-02

**Authors:** Kensuke Kataoka, Ryota Kurimoto, Hiroki Tsutsumi, Tomoki Chiba, Tomomi Kato, Kana Shishido, Mariko Kato, Yoshiaki Ito, Yuichiro Cho, Osamu Hoshi, Ayako Mimata, Yuriko Sakamaki, Ryo Nakamichi, Martin K. Lotz, Keiji Naruse, Hiroshi Asahara

**Affiliations:** ^1^Department of Systems BioMedicine, Tokyo Medical and Dental University, Tokyo, Japan; ^2^Research Fellow of Japan Society for the Promotion of Science, Tokyo, Japan; ^3^Research Core, Tokyo Medical and Dental University, Tokyo, Japan; ^4^Anatomy and Physiological Science, Tokyo Medical and Dental University, Tokyo, Japan; ^5^Department of Molecular Medicine, The Scripps Research Institute, La Jolla, CA, United States; ^6^Department of Cardiovascular Physiology, Graduate School of Medicine, Dentistry and Pharmaceutical Sciences, Okayama University, Okayama, Japan; ^7^AMED-CREST, Japan Agency for Medical Research and Development, Tokyo, Japan

**Keywords:** Mohawk, tendon, ligament, tissue engineering, mechanical-stress

## Abstract

Tendons and ligaments are pivotal connective tissues that tightly connect muscle and bone. In this study, we developed a novel approach to generate tendon/ligament-like tissues with a hierarchical structure, by introducing the tendon/ligament-specific transcription factor Mohawk (MKX) into the mesenchymal stem cell (MSC) line C3H10T1/2 cells, and by applying an improved three-dimensional (3D) cyclic mechanical stretch culture system. In our developed protocol, a combination of stable *Mkx* expression and cyclic mechanical stretch synergistically affects the structural tendon/ligament-like tissue generation and tendon related gene expression. In a histological analysis of these tendon/ligament-like tissues, an organized extracellular matrix (ECM), containing collagen type III and elastin, was observed. Moreover, we confirmed that *Mkx* expression and cyclic mechanical stretch, induced the alignment of structural collagen fibril bundles that were deposited in a fibripositor-like manner during the generation of our tendon/ligament-like tissues. Our findings provide new insights for the tendon/ligament biomaterial fields.

## Introduction

Tendons and ligaments are pivotal connective tissues that connect muscle to bone, and bone to the bone in joints. Tendons and ligaments mainly constitute the “tendon/ligament proper” and the “tendon/ligament sheath.” Tendon/ligament proper comprises highly oriented tendon/ligament cells and extracellular matrix (ECM), which contains about 70–80% of collagens and approximately 20–30% of tendon/ligament ECM associated proteins such as elastin and proteoglycans in dry mass. Tendon/ligament sheaths are connective tissues that cover tendon/ligament surfaces and contain collagen type III (Bland and Ashhurst, 1996; Killian et al., 2012; Thorpe et al., 2013; Docheva et al., 2015). Due to its ECM complexity and the poor vascularized system of the tissue, tendon/ligament injuries are one of the major issues among orthopedic diseases (Vailas et al., 1978; Liu et al., 2011; Mall et al., 2014).

To aim generating structural tendon/ligament like tissue, tissue engineering is one of promising approach. In order to recapitulate native tendon/ligament tissue structure, various methods such as cell sheets, biomaterials, and de-cellularized tissues have been developed as artificial substitutes for tendon/ligament tissue (Rodrigues et al., 2012). Recently, many studies have reported on the development of artificial tissues using cells such as mesenchymal stem cells (MSCs), embryonic tendon cells, or *Scleraxis* (*Scx*)–over-expressing embryonic stem cells (ESCs) (Kapacee et al., 2008, 2010; Chen et al., 2012; Breidenbach et al., 2015).

To construct structural tendon/ligament-like tissue via a three-dimensional (3D) culture, we focused on the developmental process of tendons and ligaments. Mechanical stress is indispensable for the growth and development of the musculoskeletal system (Dook et al., 1997; Couppé et al., 2008). For instance, mechanical stress expressing the synthesis of the cartilage ECM during development (Zuscik et al., 2008).

Furthermore, mechanical stress is essential for maintaining musculoskeletal system function. In fact, when mechanical stress is reduced, muscles show various pathologies such as atrophy (Sakuma et al., 2014).

These observations led us to investigate the synergetic effects between a 3D-culture and mechanical stress to generate structural tendon/ligament tissue. We previously showed that the tendon/ligament-specific transcription factor Mohawk (encoded by *Mkx*) is essential for the mechanical load response in tendons and ligaments. *Mkx* expression depends on mechanical stress both *in vivo* and *in vitro* and induces the expression of tendon/ligament-related genes (Kayama et al., 2016; Suzuki et al., 2016). Additionally, *MKX* differentiates the mesenchymal stem cell line C3H10T1/2 cells into tendon/ligament-like cells (Nakamichi et al., 2016).

Here, to develop a novel method to generate structural tendon/ligament-like tissue, we introduced for the first time an improved 3D cell culture and stretch system, in which various cell-stretching conditions could be adjusted. We used a stable *Mkx*-expressing C3H10T1/2 cells, which can be used for tendon/ligament-like tissue generation, as the source of tenocytes (Nakamichi et al., 2016). Combination of these strategies successfully allowed us to generate a tendon/ligament-like tissue with a highly organized collagen hieratical structure. Our findings provide new insights in the tendon/ligament biomaterial fields.

## Results

### Production of Tenocyte-Like Cells From Mesenchymal Stem Cells by *Mkx* Introduction

Preparation of tenocytes in adequate amounts is challenging because tenocyte sources and the number of tenocytes obtained from each tissue are limited. In addition, primary cultured tenocytes can easily lose their phenotype in a few passages (Yao, 2006; Shukunami, 2018). Therefore, to achieve the aim of making tendon/ligament-like tissue, we need to prepare cells that have the cell stability for cells and synthetic ability of tendon/ligament tissues. In this regard, C3H10T1/2 cells are ideal for our experiential system. It is known that the most of tendons/ligaments cells are originated from *Scx* and SRY-Box transcription factor 9 (*Sox9*) expressing progenitor cells (Sugimoto, 2013). C3H10T1/2 cells show expressing *Scx*/*Sox9* and also show MSC like multipotent differentiate capacity (Zehentner et al., 1999; Zhao et al., 2009; Shukunami, 2018).

Furthermore, previous studies reported that the tendon/ligament-specific transcription factor *Mkx* induces differentiation of the mesenchymal stem cell line C3H10T1/2 cells into abundant and uniform tenocytes-like cells (Liu, 2015; Nakamichi et al., 2016).

**FIGURE 1:**
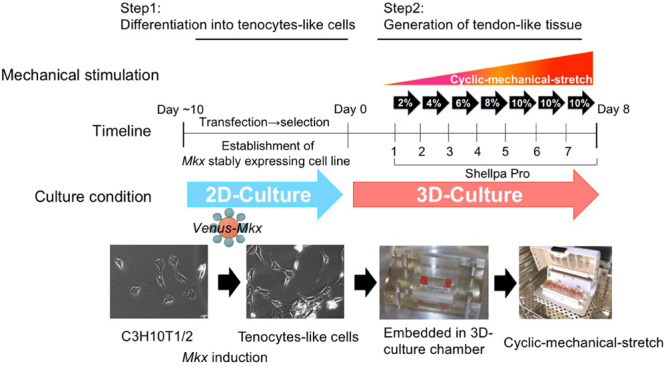
The tendon/ligament-like tissue generation protocol. Schematic illustration of the tendon/ligament-like tissue generation strategy.

Therefore, we used C3H10T1/2 cells to produce tenocytes-like cells that maintained their phenotype in the long-term. In this study, we prepared *Venus-Mkx*–expressing C3H10T1/2 cells and *Venus* (Mock)-expressing C3H10T1/2 cells as the control (Mock) (Nakamichi et al., 2016; Figure 1).

### Development of Improved Mechanical Cell Stretch System for 3D Cell Culture

Previous studies showed that mechanical stress is critical for tendon/ligament maturation (Wang, 2006; Kayama et al., 2016) and that mechanical stress under *Mkx* expression could induce critical tendon-related gene expressions (Kayama et al., 2016; Suzuki et al., 2016).

This evidence prompted us to test whether *Mkx*-expressing MSCs, combined with mechanical stress, may potentiate the generation of tendon/ligament-like tissues *in vitro*. When mechanical stress is applied to cells under two-dimensional (2D) culture conditions, cell morphology vertically orients against the cell stretching direction (Yao, 2006; Morita et al., 2013; Shukunami, 2018).

On the other hand, 3D-culture systems of tendon cells with hydrogels are recognized to provide an environment closer to that experienced by tendon cells *in vivo* (Yeung, 2015).

Thus, we utilized a 3D-culture condition to generate tendon/ligament-like tissues. *Venus-Mkx*–expressing C3H10T1/2 cells embedded in a 3D chamber by gelation of the collagen gel (Table 1). This 3D culture system aims to create an *in vivo* culture environment *in vitro*. However during long-term culture, the embedded cells undergo apoptosis because there are no blood vessels that supply nutrients and oxygen in the deep layer of this artificial culture system (data not shown). To promote cell survival under 3D and mechanical stress culture conditions (Frisch and Ruoslahti, 1997), we introduced a cocktail of pro-survival factors (Laflamme et al., 2007) into the collagen gel (Table 1 and Figure 1). We also prepared 3D-cultured *Venus* (Mock)-expressing C3H10T1/2 cells as the control.

**TABLE 1:** Tendon/ligament-like tissue 3D-culture cocktail.

**Tendon/ligament-like tissue 3D-culture protocol**	**For 1sample**	
Cellmatrix (Type1-A, nitta gelatin) (final concentration: 2 mg/mL)	32.27	ul
Collagen neutralize buffer (Type1-A, nitta gelatin) (final concentration: 1 × )	5	
0.05mg/ml Bcl-Xl BH4 4-23 (197217-1MG, Calbiochem) (final concentration: 100 nM)	0.38	
10mM Z-VAD-FMK (G723A, PROMEGA) (final concentration: 100 μM)	0.5	
0.2mg/ml Ciclosporin A (039-16301, wako) (final concentration: 400 nM)	0.12	
100ng/μl Murine IGF-1 (250-19, PeoroTech) (final concentration: 200 ng/mL)	0.1	
10mg/ml Pinacidil monohydrate (sc-203198, ChemCruz) (final concentration: 100 μM)	0.13	
5		
100 × NEAA (11140-050, gibco) (final concentration: 1 v/v%)	0.5	
100 × GlutaMAX (35050-061, gibco) (final concentration: 1 v/v%)	0.5	
100 × Penicillin/Streptomycin (15140-122, gibco) (final concentration: 1 v/v%)	0.5	
↓		
Suspending 5 × 10^5 cells into above mixture		
↓		
Gelation into geltrex (A1413302, Thermo) coated three-dimensional stretch culture chamber (STB-3.5GS, STREX) and incubated at 37°C, 5% CO2 for 60 min		
↓		
Added to the medium [final concentration of 1 × MEMα (12000-063, gibco), 10 v/v% FBS, 1 v/v% 100 × NEAA (11140-050, gibco),1 v/v% 100 × GlutaMAX (35050-061, gibco), 55 uM 2-mercaptoethanol (21985-023, gibco), 1 v/v% 100 × Penicillin/Streptomycin (15140-122, gibco)] to the chamber	1500	ul
↓		
Further incubation was carried out at 37°C, 5% CO2 for 18 h to gelation completely		

In our previous studies, we observed that that appropriate mechanical stress is beneficial for promoting tendon-related gene expression (Kayama et al., 2016; Suzuki et al., 2016).

To deliver appropriate mechanical stress on 3D-cultured tenocyte-like cells, we modified our former mechanical cell stretch system (Naruse et al., 1998). This improved mechanical cell stretch system allows us to adjust three parameters: stretch pattern (square wave, sine wave, sine wave with retention, and a combination of two types of square waves), stretch ratio [1%–20% elongation (in 1% steps)], and stretch frequency (1/600–2 Hz) (Supplementary Figure S1). Following cell embedding in the 3D-culture chamber, the samples were set to a mechanical cell stretch system. First, we have tested several different stress patterns, however, by applying too high a strain [static strain 10% at all incubation period (day 1 to day 7)] or too low a strain [static strain 2% at all incubation period (day 1 to day 7)] we were not able to generate a tissue like structure. High strain was caused to happen unintended tissue broken and low strain were not enough to accomplish collagen gel organize by embedded cell. After these testing which parameter set was best in obtaining tendon/ligament-like tissues (data not shown), we chose the following protocol: cyclic mechanical stretch was performed for one week with a gradually increasing stretch loading rate with sine wave pattern; 2% (day 1), 4% (day 2), 5% (day 3), 8% (day 4), and 10% (day 5–7) (Figure 1).

### Generation of Tendon/Ligament-Like Tissue From 3D-Cultured *Venus-Mkx*–Expressing C3H10T1/2 Cells Using Cyclic Mechanical Stretch Load

**FIGURE 2:**
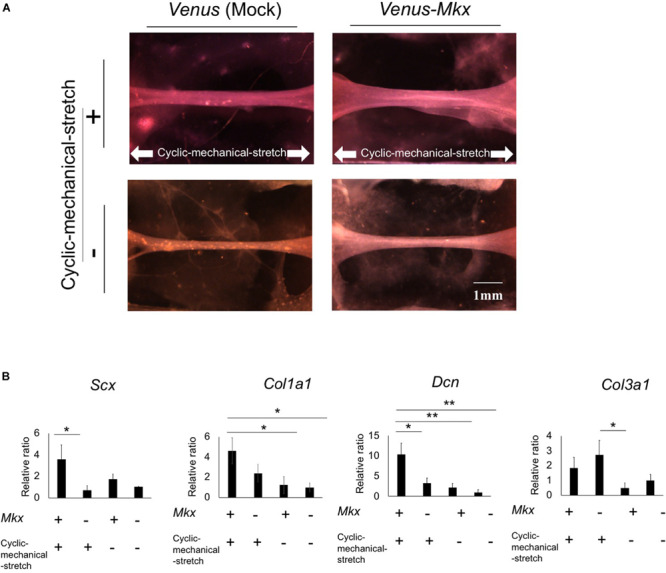
Morphology and gene expression analysis of the tendon/ligament-like tissue under four different culture conditions. **(A)** The tendon/ligament-like tissue. These tissues were generated under four different culture conditions: *Venus-Mkx*–expressing mesenchymal stem cell (MSC) line C3H10T1/2 cells undergoing cyclic mechanical stretch (right top) (VMS+), *Venus-Mkx*–expressing C3H10T1/2 cells without cyclic mechanical stretch (right bottom) (VMS–), *Venus* (Mock)-expressing C3H10T1/2 cells undergoing cyclic mechanical stretch (left top) (VS+), and *Venus* (Mock)-expressing C3H10T1/2 cells without cyclic mechanical stretch (left bottom) (VS–) (*n* = 3). The direction of the cyclic mechanical stretch load is represented by white arrows. Scale bar: 1 mm. **(B)** Quantitative real-time quantitative reverse transcription polymerase chain reaction (qRT-PCR) analysis of the expression of *Scx*, *Col1a1*, *Col3a1*, and *Dcn*. Gene expression levels are normalized to the reference gene (*Gapdh*). Error bars show the mean ± standard deviation (*n* = 3). An asterisk represents the statistical significance calculated by Bonferroni test: *p < 0.05 (for expression of *Scx*) and Tukey’s honestly significant difference (HSD) test: *p < 0.05 and **p < 0.01 (for expression of *Col1a1*, *Col3a1*, and *Dcn*).

Using the cyclic mechanical stretch conditions described above, we generated tendon/ligament-like tissues from *Venus-Mk*x–expressing C3H10T1/2 cells. To confirm the synergistic effects between *Mkx* and cyclic mechanical stretch, tendon/ligament-like tissues were generated under four different experimental conditions: *Venus-Mkx–*expressing C3H10T1/2 cells undergoing cyclic mechanical stretch (VMS+), *Venus-Mkx* without cyclic mechanical stretch (VMS−), *Venus* (Mock) *–*expressing C3H10T1/2 undergoing cyclic mechanical stretch (VS+), and *Venus* (Mock) *–*expressing C3H10T1/2 without cyclic mechanical stretch (VS−). As shown in Figure 2A, the tendon/ligament-like tissue generated in VMS+ condition is relatively thick comparing other conditions (Figure 2A). Next, to check the orientation of the cells, we performed Phalloidin staining. As a result, the orientation of actin filaments labeled with Phalloidin was observed in the stretching direction (Supplementary Figure S2).

Additionally, we examined tendon-related gene expression in each sample. The expression levels of the basic helix-loop-helix (BHLH) transcription factor *Scx*, which is highly expressed in tendon/ligament cells; collagen type I alpha 1 chain (*Col1a1*), which is the main component of tendon proper; and decorin (*Dcn*), which is involved in collagen fibrosis, were synergistically increased in the VMS + condition. Collagen type III alpha 1 chain (*Col3a1*) expression levels tend to increase depending on the cyclic mechanical stretch load but not with *Mkx* expression (Figure 2B).

### Histological Analysis of Tendon/Ligament-Like Tissue

**FIGURE 3:**
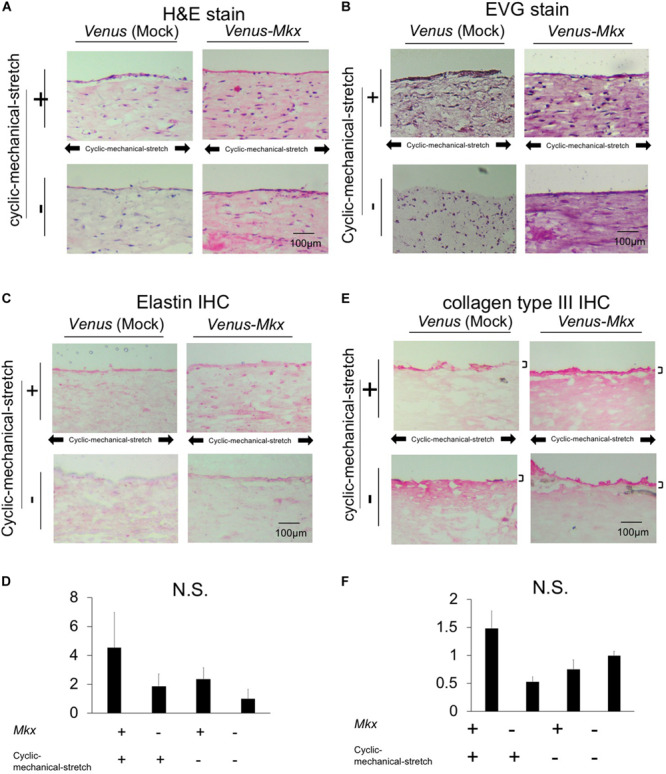
Histological and immunohistochemical analysis of the tendon/ligament-like tissue. Histological **(A,B)** and immunohistochemical (IHC) **(C,D)** analyses. These tissues were generated under four different culture conditions: VMS+ (right top), VMS– (right bottom), VS+ (left top), and VS– (left bottom) (*n* = 3). The direction of the cyclic mechanical stretch load is represented by black arrows. Scale bar: 100 μm. **(A)** Representative micrographs of hematoxylin and eosin (H&E)-stained tissue sections. **(B)** Representative micrographs of Elastica Van Gieson (EVG)-stained tissue sections. **(C)** Representative micrographs of immunohistochemical staining for elastin in each tissue section. **(D)** Relative quantitative data of panel **(C)**. **(E)** Immunohistochemical analysis of the sectioned tendon/ligament-like tissue. Representative micrographs of immunohistochemical stain of collagen type III in each tissue section. Black bars depict collagen type III staining of the surface layer of the tendon/ligament-like tissue obtained from VMS + condition (*n* = 3). Scale bar: 100 μm. **(F)** Relative quantitative data of panel **(E)**.

Histological analysis with hematoxylin and eosin (H&E) staining of the tendon/ligament-like tissue generated using VMS + condition showed that the nuclei, eosinophilic connective tissue (Figure 3A). Collagen fibers were stained in picrosirius red, and the fibers were oriented parallel to the direction of the cyclic mechanical stretch load (Supplementary Figure S3).

It has been shown approximately 70–80% of the protein of the tendon dry mass is composed of collagens, whereas approximately 1–10% of elastin (Thorpe et al., 2013), which was not included in the cell-embedding gel. These two extracellular molecules have different physical properties: collagen is rigid and elastin is elastic (Schoen and Levy, 1999).

We next tested whether the tendon/ligament-like tissues were composed of elastin by Elastica van Gieson (EVG) elastic fiber staining. The tissue generated using *Venus-Mkx*–expressing C3H10T1/2 cells showed a strong signal over the entire section (Figure 3B), whereas other samples showed relatively weak staining. Additionally, we performed elastin immunohistochemical staining on each sample and confirmed that the tissues generated by *Venus-Mkx–*expressing C3H10T1/2 cells displayed relatively strong staining (Figures 3C,D). We also performed EVG staining in a mouse achilles tendon as a comparison with our tendon like tissue. We harvested two different developmental stages from the tendon tissue: neonate (postnatal day 14: P14) and adult (3 month old: 3M). We found clear reddish fuchsin acid staining, which represents high content of collagens in the adult mouse tendon tissue proper. However, the neonate achilles tendon tissue proper showed purplish red staining, which represents a moderate amount of collagen and elastin (Supplementary Figure S4). Comparing these results, we found that mouse achilles tendon tissue, derived from the neonate, showed a similar staining pattern than our artificial tissue (VMS+ and VMS−) (Figure 3B and Supplementary Figure S4). These results suggested that *Mkx* expression and cyclic mechanical stretch cooperatively affect elastin-containing ECM remodeling during tendon/ligament-like tissue generation.

Tendons and ligaments mainly comprise the “tendon/ligament proper” and “tendon/ligament sheath. We examined whether the sheath-like structure was reproduced in the tendon/ligament-like tissues. In all samples, tissue morphology on the surface layer is different from that in the rest of the tissue. Tendon/ligament proper is composed of highly oriented tendon/ligament cells and oriented collagens, which consists of 90−95% collagen type I. On the contrary, the tendon/ligament sheath, the connective tissue that covers the tendon/ligament surface, mainly contains other type of collagens such as collagen type III (Bland and Ashhurst, 1996; Docheva et al., 2015; Marqueti et al., 2018; Taye et al., 2020). Consistent with their reports, we were able to observe the tendon sheath’s specific expression of collagen type III in mouse tendon tissue (Supplementary Figure S4).

Immunohistochemistry for collagen type III showed that VMS + C3H10T1/2 cells displayed strong and thick signal intensity at the surface layer compared with other samples (Figure 3E). VMS− and VS + condition also showed thick signal intensity at the surface of tissue but signal intensity is about less than half of VMS + condition (Figure 3F). VS− condition showed higher signal intensity comparing than VMS− and VM + but it hasn’t thick signal intensity at the surface of tissue (Figures 3E,F). This tendency was different from *Col3a1* expression in Figure 2B. Although these might indicate a discrepancy between mRNA and protein expression, it is difficult to directly comparing differences of mRNA expression level and protein tissue distribution in the section.

### Ultra-Structure Analysis of Surface and Inner Tissue of the Tendon/Ligament-Like Tissue

We found a difference in the staining patterns between the tissue surface layer and the remaining tissue from our histological analysis (Figure 3). Therefore, we further focused on these two areas. To achieve a detailed analysis tendon/ligament-like tissue morphology, we performed a scanning electron microscopy analysis.

**FIGURE 4:**
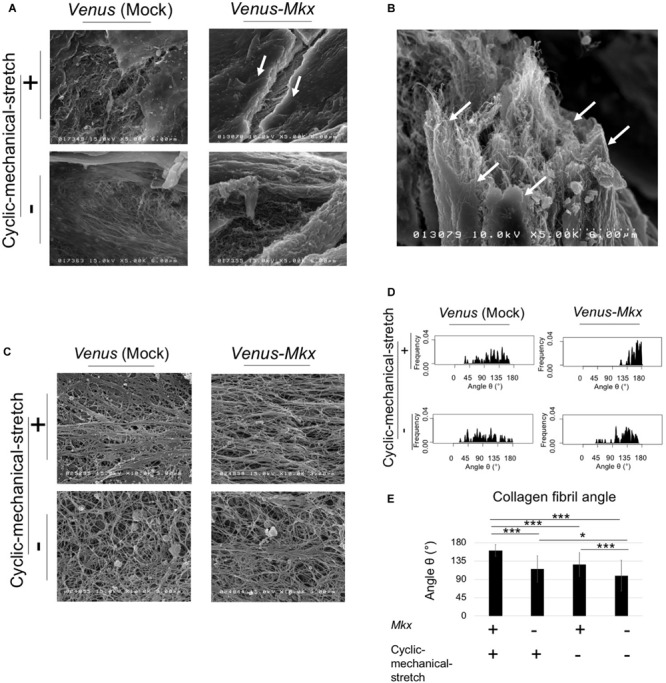
SEM analysis revealed the ultra-structure of surface and fibril arrangement of inner tissue. **(A–E)** The tendon/ligament-like tissue. These tissues were generated under four different culture conditions: VMS+ (right top), VMS– (right bottom), VS+ (left top), and VS– (left bottom). The direction of the cyclic mechanical stretch load is represented by black arrows. **(A)** Scanning electron microscopy (SEM) images of the surface layer of tendon/ligament-like tissues. The white arrow shows the surface layer of the tendon/ligament-like tissue obtained from VMS + condition (*n* = 3). Scale bar: 6 μm. **(B)** SEM image of the section illustrated in panel **(A)** taken from a different angle. The white arrow shows the surface layer of the tendon/ligament-like tissue. Scale bar: 6 μm. **(C–E)** Analysis of collagen fibril arrangement. **(C)** SEM images of the tendon/ligament-like tissue. **(D)** Kernel Density Plots of collagen fibril arrangements. Data were calculated from 60 collagen fibrils in three different fields of view. The collagen fibril angle (θ) was calculated by comparing the direction of the horizontal axis and the orientation of the collagen fiber axis. **(E)** Calculated mean collagen fibril arrangement of each sample. The collagen fibril angle (θ) was calculated by comparing the direction of the horizontal axis and the orientation of the collagen fiber axis. The mean diameter of 60 collagen fibrils from three different fields of view was calculated: for VMS + condition (right top), mean angle 161°; for VMS– condition (right bottom), mean angle 126°; VS+ condition (left top), mean angle 115°; for VS– condition (left bottom), mean angle 99°. Error bars show the mean ± standard deviation. An asterisk represents statistical significance calculated by Bonferroni test:* *p* < 0.05 and *** *p* < 0.001.

For the surface layer, the tendon/ligament-like tissue of the VMS + condition showed that the surface layer structure was uniform and thick compared with that of other samples (Figures 4A,B). An image of the same sample taken at a different angle clearly depicted the multi-layered structure of the tendon/ligament-like tissue (Figure 4B). These results suggested that *Mkx* expression and cyclic mechanical stretch have a synergistic effect in remodeling tendon/ligament sheath-like structures containing collagen type III. For the inner layer, the tendon/ligament-like tissue of VMS + condition showed a uniformly horizontal orientation of the collagen fibril compared with that of other samples (Figure 4C). We analyzed the 3D orientation of these fibrils using scanning electron microscopy and analyzed the interior of the tendon/ligament-like tissue (Figure 4C). The orientation of the collagen fibril bundle was mostly parallel to the direction of the cyclic mechanical stretch in *Venus-Mkx–*expressing C3H10T1/2 cells (Figures 4D,E).

### Analysis of Collagen Fibril Structure in the Tendon/Ligament-Like Tissue

**FIGURE 5:**
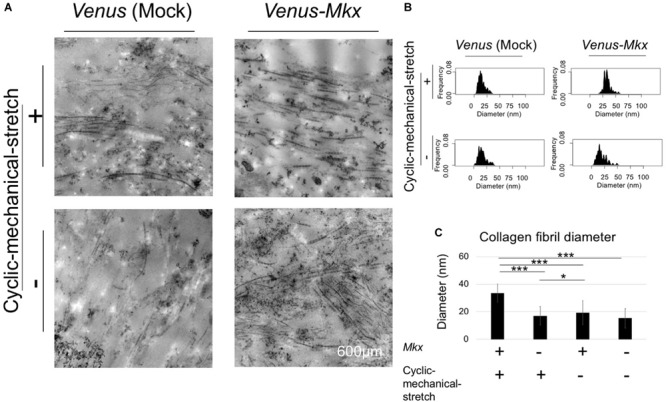
Combination of mechanical stress and *Mkx* expressing has synergistic effect for thickness of collagen fibril diameter of tendon/ligament-like tissue. **(A–C)** Analysis of collagen fibril diameter. **(A)** Vertical section of transmission electron microscopy (TEM) images of tendon/ligament-like tissue. These tissues were generated under four different culture conditions: VMS + (right top), VMS– (right bottom), VS + (left top), and VS– (left bottom) (*n* = 3). Scale bar: 600 μm. **(B)** Kernel Density Plots of collagen fibril diameters. Data were calculated from 60 collagen fibrils in three different fields of view. **(C)** Calculated mean collagen fibril diameter from each sample. The mean diameter of 60 collagen fibrils from three different fields of view was calculated: for VMS + condition (right top), mean diameter 34 nm; for VMS– condition (right bottom), mean diameter 19 nm; for VS + condition (left top), mean diameter 17 nm; for VS– condition (left bottom), mean diameter 15 nm. Error bars show the mean ± standard deviation. An asterisk represents the statistical significance calculated by Tukey’s honestly significant difference (HSD) test: *p < 0.05 and ***p < 0.001.

Next, we assessed the collagen fibril bundle diameter of the VMS + condition. Transmission electron microscopy (Figure 5A) confirmed that the diameter of the collagen fibrils increased uniformly with the cyclic mechanical stretch load (Figures 5B,C).

Altogether, these results confirmed that the cooperative effect of *Mkx* expression and cyclic mechanical stretch results not only in increasing the number of collagen fibril bundles, but also in aligning their orientation during the tendon/ligament-like tissue generation process.

### Collagen Fibril Bundle Formation in Fibripositor-Like Manner Depends on *Mkx* Expression and Cyclic Mechanical Stretch Load

The results which we have presented previously strongly suggested that the *Venus-Mkx–*expressing C3H10T1/2 cells autonomously secrete collagen fibrils and generate oriented collagen fibril bundles upon 3D cyclic mechanical stretch. Therefore, to confirm this hypothesis, we further analyzed the electron microscopy images.

If the cells are autonomously generating oriented collagen fibril bundles, the collagen fibrils in the proximity of the cells would be mature compared with those far from the cells. To test whether the cells remodel the ECM, we defined the “cell proximal region” as within 1 μm of the plasma membrane, and the “cell distal region” as greater than 1 μm from the plasma membrane; then, the length of the collagen fibril bundles in each region was measured.

**FIGURE 6:**
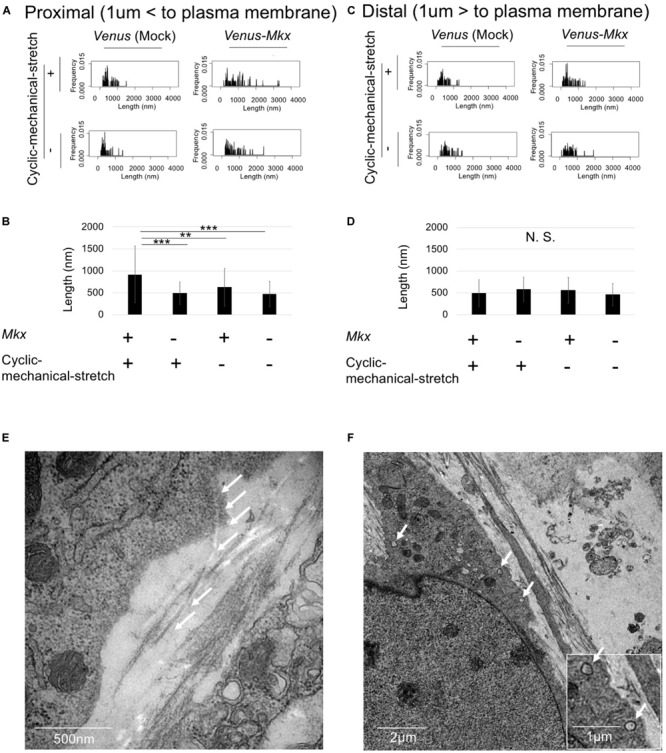
Cells autonomously remodel the extracellular matrix during tendon/ligament-like tissue generation. **(A–D)** Analysis of collagen fibril length. These tissues were generated under four different culture conditions: VMS + (right top), VMS– (right bottom), VS + (left top), and VS– (left bottom) (*n* = 2). The “cell proximal region” was defined as within 1 μm of the plasma membrane, and the “cell distal region” as more than 1 μm from the plasma membrane. The length of collagen fibril bundles in each region was measured. **(A)** Kernel Density Plots of collagen fibril length in the “cell proximal region.” Data were calculated from 60 collagen fibrils in three different fields of view. **(B)** Calculated mean collagen fibril length in the “cell proximal region” from each sample. The mean diameter of 60 collagen fibrils from three different fields of view was calculated: for VMS + condition, mean length 915 nm; for VMS– condition (right bottom), mean length 625 nm; for VS + condition (left top), mean length 495 nm; for VS– condition (left bottom), mean length 473 nm. Error bars show the mean ± standard deviation. An asterisk represents the statistical significance calculated by Bonferroni test: **p < 0.01 and ***p < 0.001. **(C)** Kernel Density Plots of collagen fibril length in the “cell distal region.” Data were calculated from 60 collagen fibrils in three different fields of view. **(D)** Calculated mean collagen fibril length in the “cell proximal region” from each sample. The mean diameter of 60 collagen fibrils from three different fields of view was calculated: for VMS + condition, mean length 489 nm; for VMS– condition (right bottom), mean length 560 nm; for VS + condition (left top), mean length 495 nm; for VS– condition (left bottom), mean length 460 nm. Error bars show the mean ± standard deviation. Statistical significance calculated by one-way analysis of variance (ANOVA). **(E,F)** Deposition of collagen fibrils in a fibripositor-like manner (*n* = 2). **(E)** Direct secretion of collagen fibrils through the plasma membrane into the extracellular matrix. The white arrow indicates the collagen fibril. Scale bar: 500 nm. **(F)** Intracellular lumen of the fibripositor-like structure. The white arrow indicates the fibripositor-like lumen structure. Scale bar: 2 μm. The bottom panel shows a higher magnification of the fibripositor-like lumen structure. The white arrow indicates fibripositor-like lumen structure. Scale bar: 1 μm.

The results showed that the lengths of the collagen fibril bundles were longer in the proximal area of the cell than they were far from the cell (Figures 6A–D). Furthermore, statistical analysis of the collagen fibril bundle length in the “cell proximal region” revealed significantly longer bundles (Bonferroni test; *p* < 0.01 compared with the VMS− condition, *p* < 0.001 compared with the VS + condition and VS− condition) in VMS + condition than those in the other samples (Figure 6B). These results suggest that *Mkx* expressing and applying mechanical stretch have a synergistic effect with regard to collagen length (Figures 6A–F).

To determine the mechanisms of collagen maturation in *Venus-Mkx–*expressing C3H10T1/2 cells during cyclic mechanical stretch, we analyzed collagen secretion. A detailed analysis of the transmission electron microscopy images of VMS + C3H10T1/2 cells showed that the cells secrete mature collagen bundles directly into the plasma membrane (Figure 6E), which resembles fibripositors (Canty et al., 2004). A previous report showed that cellular fibripositor protrusions contain collagen fibrils (Canty et al., 2004; Kapacee et al., 2008). Interestingly, the VMS + C3H10T1/2 cells showed a typical fibripositor lumen-like structure (Figure 6F).

These results revealed that, in samples subjected to cyclic mechanical stretch and stably express *Mkx*, the cells autonomously secrete collagen fibril bundles in a fibripositor-like manner.

## Discussion

In this study, we developed a novel artificial tendon/ligament-like tissue generation method by coupling the expression of the tendon/ligament-specific transcription factor MKX with a 3D cyclic mechanical stretch culture system. Specifically, we embedded cells that stably express *Mkx* in a collagen gel and cultured them in three dimensions under a gradually increasing cyclic mechanical stretch load to mimic tendon/ligament development conditions.

However, how to generate structures that closely mimic the ECM in the *in vivo* tendon/ligament environment, which comprise several ECM-related proteins, such as collagen type I, collagen type III, and elastin (Thorpe et al., 2013; Docheva et al., 2015), could not be established.

To overcome these difficulties, we used cells that were stably transfected with a vector expressing the transcription factor MKX, which has the ability to promote collagen maturation and arrangement. The analysis of our electron microscope images confirmed that horizontal orientation of the collagen fibril bundles occurred exclusively in the cells that stably expressing *Mkx* and were subjected to cyclic mechanical stretch. The oriented collagen fibrils were secreted in a fibripositor-like manner similar to specific collagen secretion in the embryonic tendon development process (Canty et al., 2004). This phenotype was not observed in the control sample [*Venus* (Mock)-expressing C3H10T1/2 cells] or in samples not subjected to cyclic mechanical stretch, demonstrating the synergistic effects between MKX and cyclic mechanical stretch on collagen fibril bundle secretion and remodeling processes.

Importantly, we confirmed the presence of an organized ECM structure in the tendon/ligament-like tissue, similar to that of actual tendon tissues. The result of EVG staining showed that our artificial tissue (VMS+ and VMS−) has a similar staining pattern to neonatal mouse achilles tendon tissue (Figure 3B and Supplementary Figure S4). Analysis of the tendon/ligament-like tissue section confirmed that collagen type III aligned to the surface layer and that the tendon proper contained elastin. This organized ECM was rarely observed in the control sample [*Venus* (Mock)-expressing C3H10T1/2 cells] or in samples without cyclic mechanical stretch, which confirmed the synergistic effects between MKX and cyclic mechanical stretch in the remodeling and organization of the ECM. Previous reports have shown that collagen type III is specifically expressed in the tendon sheath, suggesting that collagen type III is among the “morphological marker proteins” of the tendon and ligament tissue (Docheva et al., 2015; Marqueti et al., 2018; Taye et al., 2020). Consistent with previous reports, we could observe the tendon sheath’s specific expression of collagen type III in mouse tendon tissue (Supplementary Figure S4). We also observed the specific collagen type III distribution in the tendon/ligament-like tissue surface layer (Figure 3E), suggesting that the surface layer of an artificial tendon might possess tendon sheath characters.

The cell senses mechanical stress via a specific mechanical sensing receptor and a mechanical stress specific signal transduction. A previous report showed that *Mkx* and its upstream GTF2IRD is involving tendon mechanical sensing (Kayama et al., 2016). It has also been shown that MKX has the ability to maintain tenocyte identity via repressed cartilage specific transcription factor SOX9 (Suzuki et al., 2016). These reports indicate that *MKX* has dual role in tendon development: respond to the mechanical stress and maintain tendon tissue. This dual effect of *MKX* might be involved in generating the most constructive structure at the VMS+ condition.

With an aim to recapitulate the *in vivo* mechanical environment, various cell stretching devices have been developed and marketed (Naruse et al., 1998; Matheson et al., 2006; Chen et al., 2012; Kreutzer et al., 2014; Mihic et al., 2014; Breidenbach et al., 2015). In this study, we improved on our previously published mechanical cell stretch system (Naruse et al., 1998). The advantage of this new system is that it allows the configuration of detailed stretching conditions by setting three main parameters (1) stretch pattern, (2) stretch ratio, and (3) stretch frequency. By altering the stretch pattern (square wave, sine wave, sine wave with retention, or a combination of two types of square waves); stretch ratio [1–20% elongation (in 1% steps)]; and stretch frequency (1/600–2 Hz), this system could be configured to mimic the dynamic environment inside the human body and could be used to generate other tissues/organs requiring cyclic mechanical stretch.

In summary, we developed a novel system and method to generate structural tendon/ligament-like tissues containing elastin, oriented collagen type III, and collagen fibril bundles deposited in a fibripositor-like manner. By changing the shape of the 3D-culture chamber, it could be possible to prepare various fibrous tissues such as intervertebral disc-like artificial tissue and rotator cuff-like artificial tissue.

Our findings provide new insights in the tendon/ligament biomaterial fields.

## Materials and Methods

### Cell Culture and Retrovirus Infection

C3H10T1/2 culture, retrovirus transfection, and establishment of *Venus-* (Nagai et al., 2002) or *Venus-Mkx* C3H10T1/2 cells were performed as previously described (Nakamichi et al., 2016). Briefly, C3H10T1/2 cells were cultured in alpha minimal essential medium (MEMα) with 10 v/v% fetal bovine serum (FBS) and 1 v/v% penicillin/streptomycin (15140-122, Gibco, MA, United States). Retrovirus infection was performed in the supernatant of retrovirus vector (pMIGR/*Venus*, pMIGR/*Venus-Mkx*) transfected PLAT-E cells. In order to clearly observe the cell shape, we simultaneously introduced *mCherry* in both cell lines using the supernatant of pMIGR/*mCherry*-transfected PLAT-E cells. Stable cell lines were established by 1 μg/mL puromycin (ant-pr-1, InvivoGen, CA, United States) and 10 μg/mL blasticidin S (026-18711, FUJIFILM Wako Pure Chemical Corp., Osaka, JAPAN) selection for 1 week.

### Isolation of Mouse Tendon and Fixation

Achilles tendons were harvested from neonate (postnatal day 14: P14) or adult (3 month old: 3M) C57BL/6N mice (Sankyo Labo Service Corporation, Tokyo, Japan). All mice were kept in specific pathogen-free facilities. After euthanizing anesthetized mice through cervical dislocation, both achilles tendons were removed. All tendons were fixed with 4% paraformaldehyde, dehydrated with 30% sucrose and embedded with OCT (Sakura Finetek, Torrance, CA, USA), and frozen at −80°C before section. All animal experiments were performed according to protocols approved by the Institutional Animal Care and Use Ethical Committee at the Tokyo Medical and Dental University (Approval No. A2018-096A).

### 3D-Culture

The cells were embedded in a 3D-culture cocktail (Table 1). The 3D-culture cocktail was constructed by mixing collagen gel [final concentrations: 2 mg/mL Cellmatrix (Type I-A, Nitta Gelatin Inc., Osaka, JAPAN) and 1 × collagen neutralization buffer (Type I-A, Nitta Gelatin Inc.)], pro-survival cocktail according to Laflamme et al. (2007) final concentrations: 100 nM B-cell lymphoma extra-large (Bcl-Xl) BH4 4-23 (197217-1MG, Calbiochem), 100 μM carbobenzoxy-valyl-alanyl-aspartyl-[O-methyl]-fluoromethylketone (Z-VAD-FMK) (G723A, Promega, WI, United States), 400 nM cyclosporin A (039-16301, FUJIFILM Wako Pure Chemical Corp.), 200 ng/mL murine insulin-like growth factor 1 (IGF-1) (250-19, PeproTech, NJ, United States), and 100 μM pinacidil monohydrate (sc-203198, ChemCruz, TX, United States), and medium [final concentration: 1 × MEMα (12000-063, Gibco), 10 v/v% FBS (2916154, MP Biomedical), 1 v/v% 100 × non-essential amino acid solution (NEAA) (11140-050, Gibco), 1 v/v% 100 × GlutaMAX (35050-061, Gibco), and 1 v/v% 100 × penicillin/streptomycin (15140-122, Gibco)].

To avoid irregular adhesion between the bottom of the 3D chamber and the 3D-culture cocktail, the bottom of the 3D stretch culture chamber (STB-3.5GS, STREX Inc., Osaka, JAPAN) was coated with 40 μL of Geltrex (A1413302, Thermo, MA, United States) and incubated at 37°C, 5% CO_2_ for 30 min to allow Geltrex gelation. This step is important because irregular adhesion with the 3D chamber may hinder the structuring of the tendon/ligament-like tissue. The 3D-culture cocktail and cell mixture were transferred to the Geltrex-coated 3D stretch culture chamber and incubated at 37°C, 5% CO_2_ for 60 min for gelation. Following gelation, MEMα medium containing 10 v/v% FBS, 1 v/v% 100 × penicillin/streptomycin (15140-122, Gibco), 1 v/v% 100 × GlutaMAX (35050-061, Gibco), 1 v/v% 100 × NEAA (Gibco 11140 - 050), and 55 μM 2-mercaptoethanol (21985-023, Gibco) was added to the chamber. Further incubation was carried out at 37°C, 5% CO_2_ for 18 h for complete gelation.

### Mechanical Stimulation

Following gelation, the 3D-cultured samples were set into a mechanical cell stretch system device (Shellpa Pro, Menicon Co., Ltd./Life Science Department, Aichi, Japan). Cyclic mechanical stretch was performed for one week, with a gradually increasing stretch loading rate to mimic the tendon/ligament development process: 2% (day 1), 4% (day 2), 5% (day 3), 8% (day 4), and 10% (day 5–7). The stretch loading rate was defined according to the following formula.

Stretch loading rate (%) = (width of the 3D stretch culture before stretching – width of the 3D stretch culture after stretching)/(width of the 3D stretch culture before stretch × 100). The cyclic mechanical stretch was programed at 0.25 Hz for 18 h/day, followed by resting for 6 h/day at 37°C, 5% CO_2_. Samples which were appropriately anchored with a 3D chamber sponge and which had no irregular adhesion to the chamber side-wall were assayed in this study.

### Histological and Immunohistochemical Analysis

Tendon/ligament-like tissue or mouse achilles tendon was fixed in 4% paraformaldehyde overnight at 4°C, washed in 1 × phosphate-buffered saline (PBS), cryo-preserved in 20% sucrose overnight, and embedded in optimal cutting temperature (OCT) compound (45833, Sakura Finetek Japan Co., Ltd., Tokyo, Japan). Then, the tendon/ligament-like tissue was cryo-sectioned at 10 μm and desiccated by air-drying overnight. Histological staining using hematoxylin (131-09665, FUJIFILM Wako Pure Chemical Corp.) and eosin (051-06515, FUJIFILM Wako Pure Chemical Corp.), Picrosirius red staining kit (24901-500, Polysciences, Inc., PA, United States), and Elastica Van Gieson (EVG) staining kit (1.15974.0002, Merck Millipore, Burlington, MA, United States) was performed according to the manufacturers’ instructions. Immunohistochemical staining was performed using a Vectastain ABC-AP Rabbit IgG Kit (AK-5001, VECTOR LABORATORIES, INC., CA, United States) and Vector Red (SK-5100, VECTOR LABORATORIES, INC.) according to the manufacturer’s instructions. Anti-Collagen III (1/500 dilution) (ab7778, Abcam plc, Cambridge, United Kingdom) and Anti-Elastin antibodies (1/500 dilution) (ab217356, Abcam plc) were used as the primary antibodies. We quantified the images using Image J software (NIH). In brief, the image was split into three colors (red, green, and blue), and only red colored images were picked up (red − blue). All images stained and photographed at the same time were set to the same threshold. The relative ratio of the area that exceeded the threshold from three different fields of view was calculated. To stain F-Actin, Alexa Fluor 594-phalloidin (A12381, Life technology) staining was performed according to the manufacturer’s instructions. DAPI (VECTASHIELD with DAPI, H-1200, funakoshi) was used for nuclear staining.

### RNA Isolation and qRT-PCR

RNA was isolated in ISOGEN (319-90211, NIPPON GENE CO., LTD., Toyama, Japan) using a teflon homogenizer and was reverse-transcribed using a ReverTra Ace (TRT-101, TOYOBO CO., LTD., Osaka, Japan) according to the manufacturer’s instructions. Complementary DNA was quantitated by qRT-PCR using a Thunderbird SYBR mix (QPS-201, TOYOBO CO., LTD.). *Gapdh* expression served as the control for mRNA expression. Changes in gene expression were quantified using the ΔΔCT method (Livak and Schmittgen, 2001). Primer sequences are listed in Supplementary Table S1.

### Statistical Analysis and Image Quantification

All statistical analyses were performed using R version 3.4.3 (R Core Team, 2017). First, significance of variance among samples was calculated by Bartlett’s test using the command “bartlett.test.” Secondly, if the samples had equal variance, one-way ANOVA was conducted using the command “aov,” if samples had no equal variance, Kruskal-Wallis rank sum test was conducted using the command “kruskal.test.”

Finally, if the samples rejected the null hypothesis of one-way ANOVA, the significant of each sample was calculated with *post hoc* comparisons by Tukey’s honest significant difference (HSD) test using the command “TukeyHSD,” and if the samples rejected the null hypothesis of the Kruskal-Wallis rank sum test, the significance of each sample was calculated with *post hoc* comparisons by Bonferroni using the command “pairwise.*t*.test (p.adjust.method = “bonferroni”).” *p* < 0.05 was considered significant in all statistical analyses. The command “density” was used to calculate and depict the Kernel Density Plots. The diameter and arrangement of collagen fibrils were analyzed using the ImageJ software (Schneider et al., 2012). The collagen fibril angle (θ) was calculated by comparing the direction of the horizontal axis and the orientation of the collagen fiber axis.

### Electron Microscopy

Tendon/ligament-like tissues were dissected and fixed in 2.5% glutaraldehyde in 0.1 M phosphate buffer (PB) overnight. For transmission electron microscopy (TEM), the specimens (*n* = 3) were washed with 0.1 M PB, post-fixed in 1% osmium buffered with 0.1 M PB for 2 h, and dehydrated in a graded series of ethanol. Then, the specimens were embedded in Epon 812, sliced into ultrathin sections (70 nm), collected on copper grids, and double-stained with uranyl acetate and lead citrate. The specimens were observed using TEM (H-7100, Hitachi, Ltd., Tokyo, Japan). For scanning electron microscopy (SEM), the specimens (*n* = 2) were dried in a critical-point drying apparatus (HCP-2, Hitachi, Ltd.) with liquid CO_2_ and were spatter-coated with platinum. Then, the specimens were observed using SEM (S-4500, Hitachi, Ltd.).

## Data Availability Statement

All the data required to reproduce this study are included in this published article and Supplementary Information. The raw data required to reproduce these findings are available from the corresponding author on reasonable request.

## Ethics Statement

The animal study was reviewed and approved by the Institutional Animal Care and Use Ethical Committee at the Tokyo Medical and Dental University (Approval No. A2018-096A).

## Author Contributions

KK, TC, YI, RN, ML, and HA designed all of the experiments. KN contributed refinement of the mechanical cell stretch system and advised on tendon/ligament-like tissue generation. KK performed the 3D-culture with cyclic mechanical stretching, qRT-PCR of tendon/ligament-like tissue, and all statistical analyses. KK, RK, HT, TK, KS, and MK performed all the histological/immuno-histochemical analyses. AM and YS performed the sample preparation of transmission/scanning electron microscope. KK, RK, HT, TK, KS, MK, YC, and OH performed the analysis of the scanning electron microscope. KK, RK, HT, TK, and KS performed the analysis of the transmission electron microscope. KK, TC, and HA interpreted all the experimental data and wrote the manuscript. ML and HA supervised the laboratory and all experiments.

## Conflict of Interest

The authors declare that the research was conducted in the absence of any commercial or financial relationships that could be construed as a potential conflict of interest.
