# Supplementary material for: In vitro Neo-Genesis of Tendon/Ligament-Like Tissue by Combination of Mohawk and a Three-Dimensional Cyclic Mechanical Stretch Culture System
Source: Front Cell Dev Biol. 2020 Jun 2;8:307. doi: 10.3389/fcell.2020.00307 (PMC7326056; doi:10.3389/fcell.2020.00307)
Supplement: Supplementary file 1 [file Data_Sheet_1.docx]

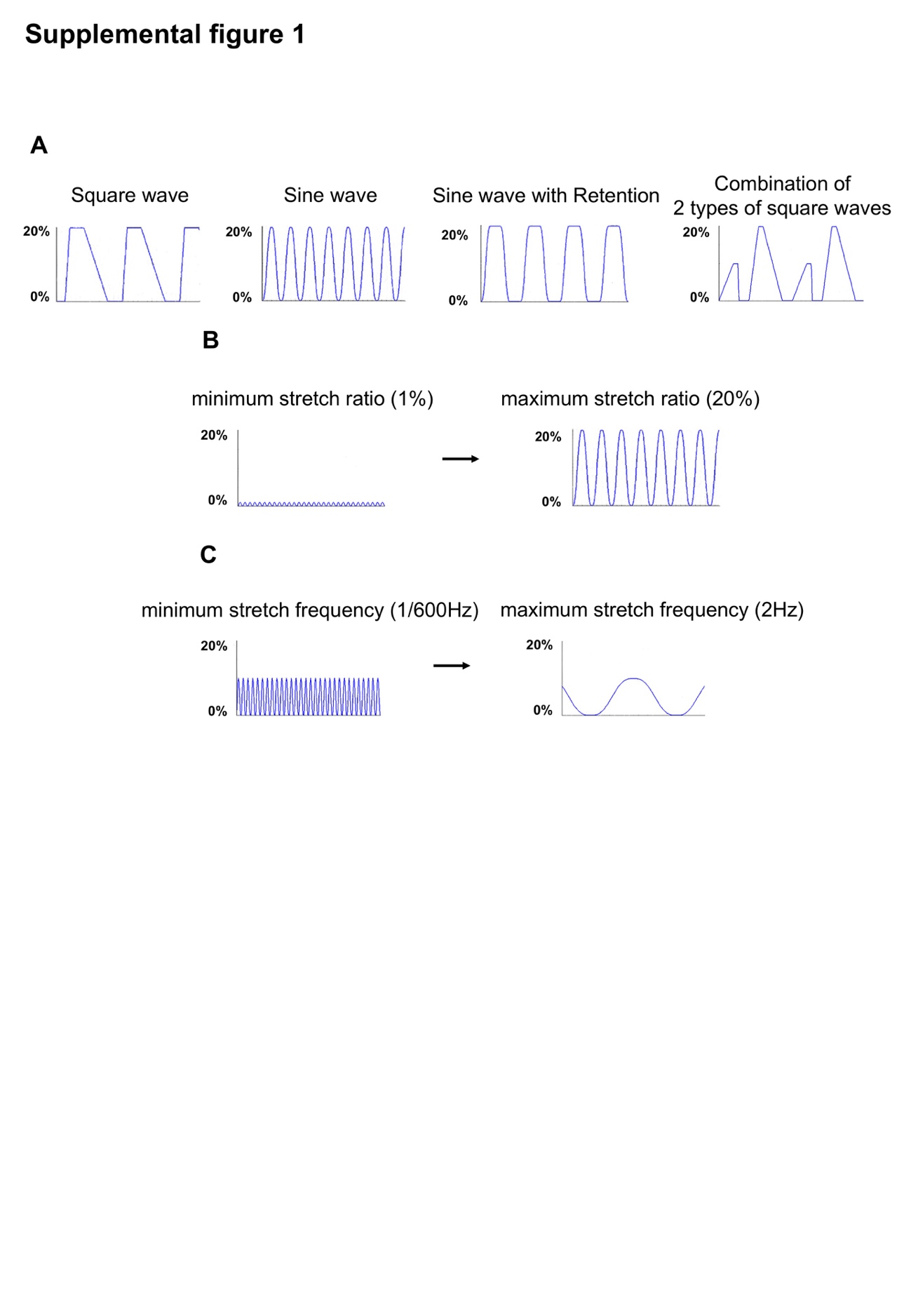


**Fig. S1 Improved mechanical cell stretch system allows fine tuning of various mechanical stretch-related parameters**

(A–C) Cell stretching parameters of our improved mechanical cell stretch system . (A) Stretch pattern of square wave, sine wave, sine wave with retention, combination of 2 types of square waves. (B) Minimum stretch ratio (1%) and maximum stretch ratio (20%). (C) Minimum stretch frequency (1/600 Hz) and maximum stretch frequency (2 Hz) are shown.


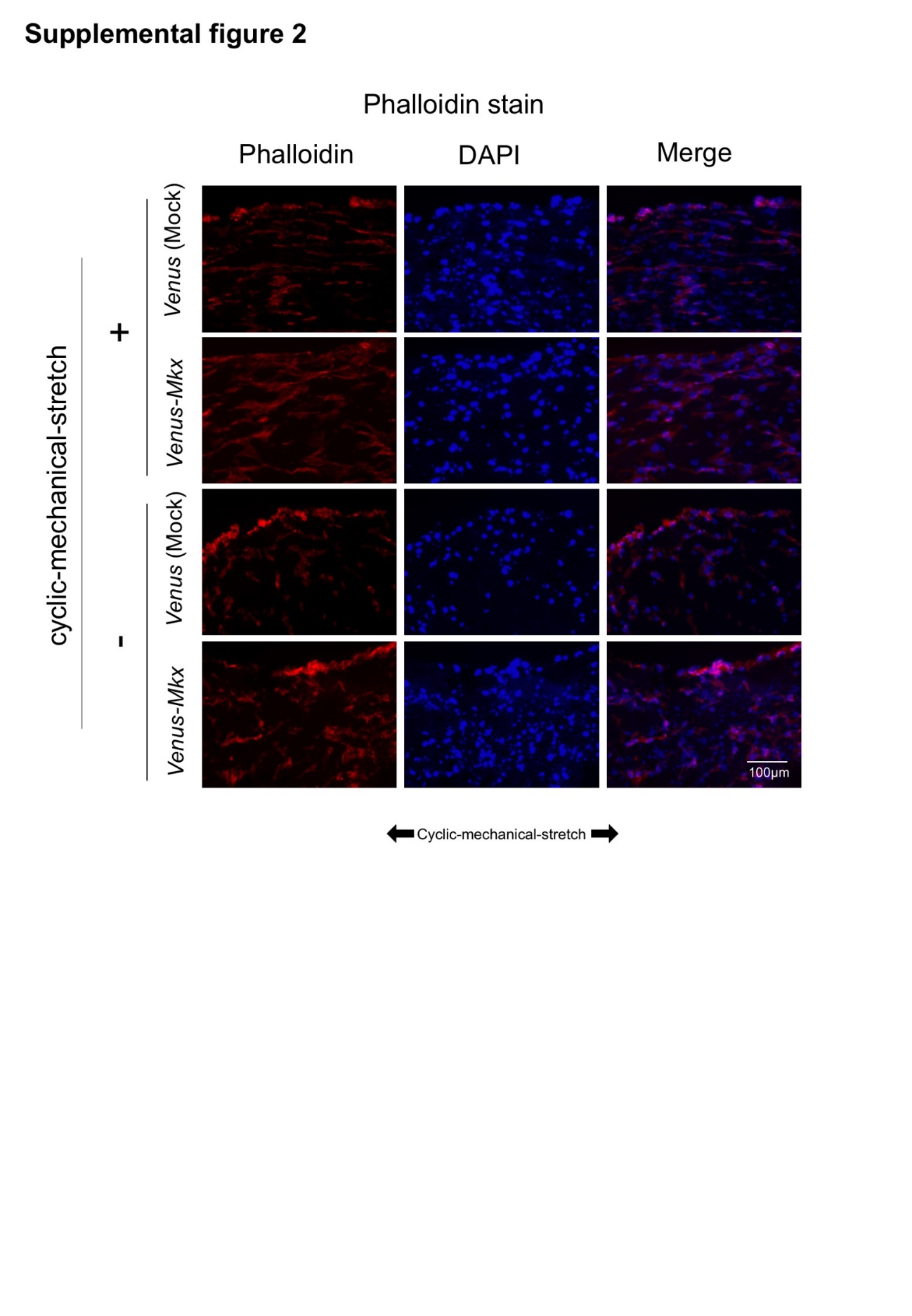


**Fig. S2 Phalloidin staining of the tendon-like tissue**

Representative micrographs of phalloidin/DAPI staining tissue sections. These tissues were generated under four different culture conditions: VMS+ (right top), VMS- (right bottom), VS+ (left top), and VS- (left bottom) (n = 1). The direction of the cyclic mechanical stretch load is indicated by black arrows. Scale bar: 100 μm.


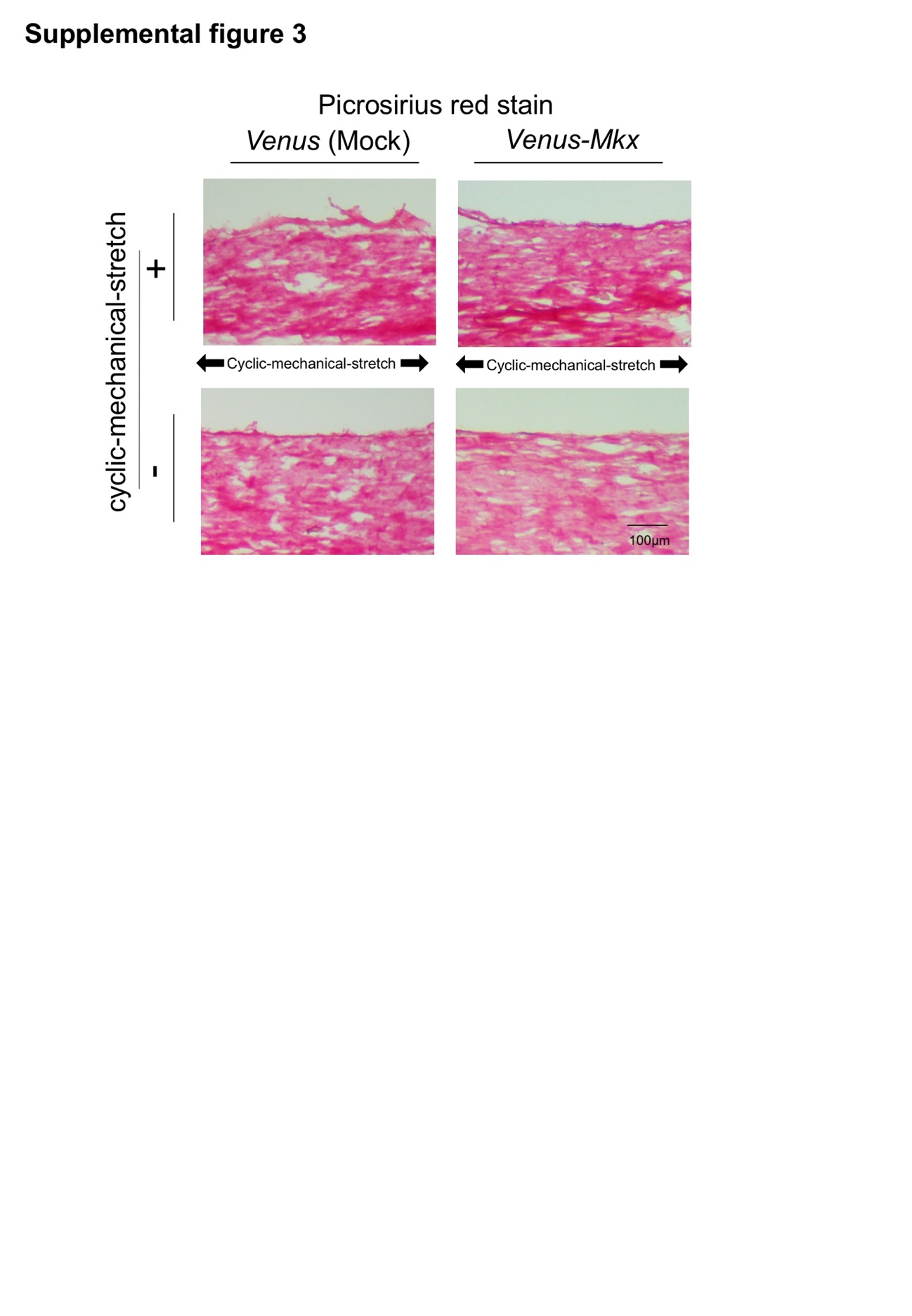


**Fig. S3 Picrosirius red staining of the tendon-like tissue**

Representative micrographs of picrosirius red-stained tissue sections. These tissues were generated under four different culture conditions: *Venus-Mkx*–expressing mesenchymal stem cell (MSC) line C3H10T1/2 cells undergoing cyclic mechanical stretch (right top) (VMS+), *Venus-Mkx*–expressing C3H10T1/2 cells without cyclic mechanical stretch (right bottom) (VMS-), *Venus* (Mock)-expressing C3H10T1/2 cells undergoing cyclic mechanical stretch (left top) (VS+), and *Venus* (Mock)-expressing C3H10T1/2 cells without cyclic mechanical stretch (left bottom) (VS-) (n = 3). The direction of the cyclic mechanical stretch load is indicated by black arrows. Scale bar: 100 μm.


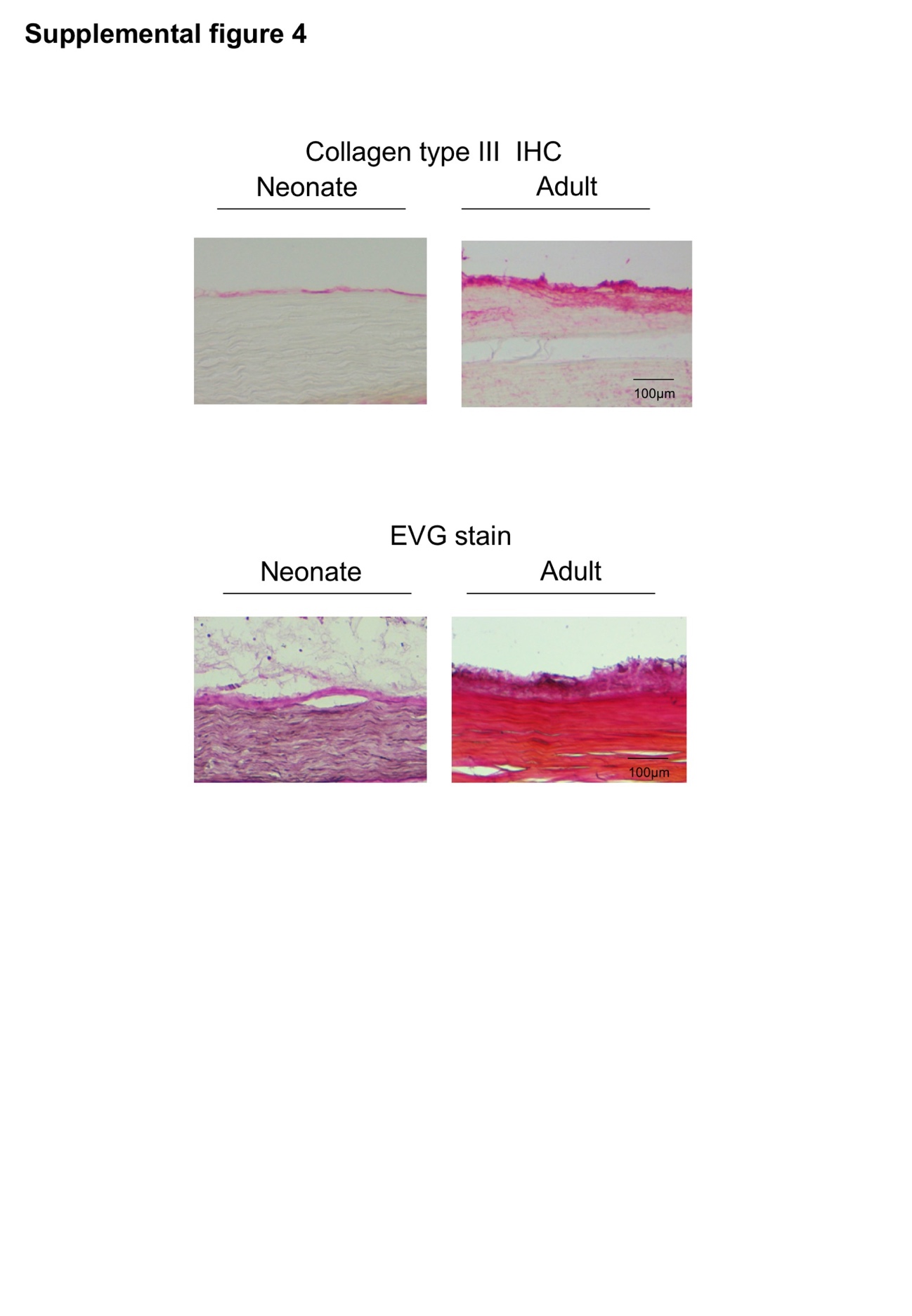


**Fig. S4 histological analysis of the native mouse tendon tissue**

Representative micrographs of Representative micrographs of Elastica Van Gieson (EVG) staining and immunohistochemical staining of collagen type III using mouse achilles tendon tissue section harvested from neonate (postnatal day 14: P14) or adult (3month old: 3M) (n = 1). Scale bar: 100 μm.


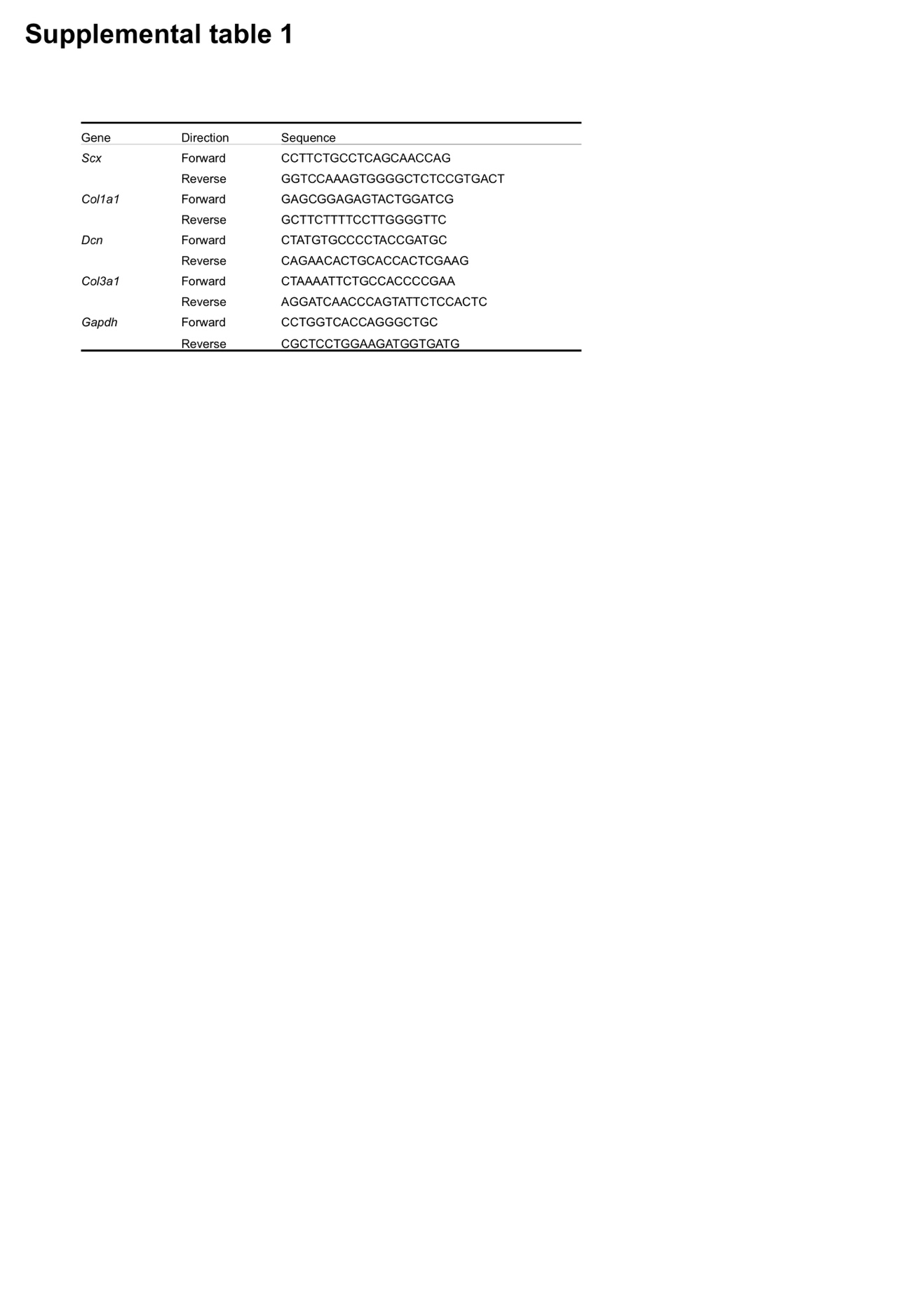


**Table S1 Primer sequences for tendon-like tissue qRT-PCR**
